# The diagnostic value of miR-4281 for knee osteoarthritis and its mechanism of action on the extracellular matrix of chondrocytes and apoptosis

**DOI:** 10.1186/s41065-026-00711-9

**Published:** 2026-07-25

**Authors:** Wei Song, Qin Yu, Baohui Zhao, Yu Zhang, Zhigang Kong

**Affiliations:** 1 Department of Orthopedics, Zhangzhou Affiliated Hospital of Fujian Medical University, Zhangzhou, 363000 China; 2 Department of Orthopedics, West China Hospital, Sichuan University, Sichuan, 610041 China; 3 West China School of Nursing, Sichuan, 610041 China; 4 Department of Comprehensive Orthopedics, Affiliated Hospital of Hebei University, Hebei, 071000 China; 5 Department of Day Treatment Center, The Third Affiliated Hospital of Naval Medical University, PLA, Shanghai, 201185 China; 6 Changzhou Wujin People’s Hospital, No.2 Yongning North Road, Tianning District, Changzhou, 213000 China

**Keywords:** Knee osteoarthritis, miR-4281, BCL3, Extracellular matrix, Apoptosis

## Abstract

**Background:**

The destruction of tissues such as bone and subchondral bone is an important pathological feature of knee osteoarthritis (KOA).

**Aim:**

To explore the diagnostic value of serum miR-4281 and its mechanisms influencing KOA.

**Materials and methods:**

The mRNA levels of miR-4281, SOX9, COL2A1, MMP3, MMP13 and BCL3 were detected by RT-qPCR. Pearson correlation was used to assess the correlation between serum miR-4281 and WOMAC score or BCL3. The receiver operating characteristic (ROC) curve was used to analyse the diagnostic efficacy of miR-4281 for KOA. Multivariate Logistic regression was used to assess the impact of miR-4281 on KOA. The dual luciferase reporter gene assay demonstrated the relationship between miR-4281 and BCL3. Annexin V-FITC/PI Apoptosis Kit was used to assess the apoptosis level of C28/I2 cells.

**Results:**

In KOA patients, serum miR-4281 was significantly upregulated, while BCL3 was markedly downregulated. miR-4281 exhibited a positive correlation with the WOMAC score and demonstrated high diagnostic efficacy for KOA. 10 ng/mL IL-1β induced the expression of miR-4281, MMP3, and MMP13 in C28/I2 cells, as well as cell apoptosis, while inhibiting the expression of SOX9, COL2A1 and BCL3. miR-4281 inhibitors promote SOX9 and COL2A1 expression while suppressing MMP3 and MMP13 expression as well as apoptosis. Silencing BCL3 reversed the mitigating effects of miR-4281 inhibitors on IL-1β-induced extracellular matrix (ECM) degradation and apoptosis in C28/I2 cells.

**Conclusion:**

miR-4281 has a good diagnostic efficacy for KOA and it alleviates the ECM degradation and apoptosis of chondrocytes induced by IL-1β by targeting BCL3.

**Supplementary Information:**

The online version contains supplementary material available at https://doi.org/10.1186/s41065-026-00711-9.

## Introduction

Knee osteoarthritis (KOA) is a chronic degenerative disease characterised by articular cartilage degeneration, synovial inflammation, and osteophyte formation [1]. Its pathological core manifestation is the progressive destruction of articular cartilage and loss of joint function [2]. The prevalence of this condition increases significantly among the elderly population, affecting approximately 9.6% of men and 18% of women globally [3]. As the population ages, the disease burden continues to intensify. The cause of KOA involves the combined effects of multiple factors, such as abnormal mechanical stress, release of inflammatory factors, and genetic susceptibility, which lead to a metabolic imbalance in chondrocytes [4, 5]. Currently, the main clinical treatment methods include the use of non-steroidal anti-inflammatory drugs to relieve pain, joint cavity injections to improve symptoms, and surgical interventions such as joint replacement [6, 7]. However, existing methods face limitations in reversing cartilage damage, alongside concerns regarding drug side effects, surgical trauma, and long-term complications. In terms of diagnosis, imaging examinations such as X-rays and MRI, as well as joint fluid analysis, are the main tools [8]. Imaging examinations exhibit lower sensitivity in the early stages of disease, making it difficult to detect minor cartilage lesions at an early stage [9]. Although synovial fluid analysis can assist in diagnosis, it constitutes an invasive procedure with low patient acceptance. Therefore, the lack of specific early diagnostic markers has led to some patients being diagnosed at an advanced stage (mid-to-late stage), thus missing the best intervention opportunity.

The destruction and degradation of articular cartilage constitute the core pathological mechanism in the development of KOA, and represent the primary cause of joint dysfunction [10]. The severity of this pathology directly determines the progression and prognosis of KOA. Cartilage, as a buffering structure of joints, relies on the homeostasis of extracellular matrix (ECM) synthesised by chondrocytes for its integrity [11]. ECM is primarily composed of collagen and proteoglycans, providing mechanical support and lubrication for joints [12]. In KOA, inflammatory factors and mechanical damage lead to a reduction in the synthesis of ECM by chondrocytes and an increase in the activity of degrading enzymes, causing the loss of matrix components and thereby accelerating cartilage degeneration [13]. Concurrently, this process is also closely associated with chondrocyte apoptosis. The ECM disruption impairs the signals that maintain cell viability, and the apoptotic chondrocytes further release pro-inflammatory mediators, thus creating a vicious cycle [14]. Research indicates that chondrocyte apoptosis not only directly reduces cell numbers but also exacerbates joint damage by activating downstream inflammatory pathways, thereby constituting a pivotal step in the progression of knee osteoarthritis [15]. Therefore, regulating the ECM homeostasis and apoptotic process of chondrocytes has become an important target for intervening in the progression of KOA.

MicroRNAs (miRNAs), as crucial regulators of gene expression, play a key role in osteoarthritis by targeting mRNAs to influence cell proliferation, apoptosis, and ECM metabolism [16]. For instance, miR-214-3p delays cartilage degradation by regulating the expression of type II collagen (COL2A1) and matrix metalloproteinases (MMPs) [17]. The miR-141/200c inhibitor exhibits a potent protective effect on cartilage [18]. For miR-4281, there is a study reporting that its expression is significantly elevated in Tregs of patients with rheumatoid arthritis [19]. It is well known that both rheumatoid arthritis and osteoarthritis cause joint pain, swelling, stiffness and restricted movement, and both can trigger severe inflammatory responses. Therefore, we speculate that miR-4281 may also play an important role in the occurrence and development of osteoarthritis. However, the specific mechanism of its action in both rheumatoid arthritis and osteoarthritis has not been reported yet. Therefore, this study focuses on miR-4281, aiming to explore its value as a diagnostic biomarker for KOA and elucidate its mechanism of influencing disease progression by regulating chondrocyte ECM homeostasis and apoptotic pathways.

## Materials and methods

### Patients

A cohort of 126 KOA patients admitted to West China Hospital, Sichuan University, was selected as the disease group (KOA). Additionally, 126 healthy individuals who underwent physical examinations at our hospital during the same period were selected in a 1:1 ratio to serve as the healthy control group (Health). Inclusion criteria: (1) Patients must be over 18 years old, meet the diagnostic criteria for KOA, and have normal organ functions. (2) The patient’s K-L classification is at levels II to IV, and there are clear bone spurs, multiple bone spurs accompanied by moderate narrowing of the joint space, huge bone spurs accompanied by severe narrowing of the joint space, and subchondral bone sclerosis. (3) The patient’s coagulation function was normal and no relevant medications had been taken before the sample collection. Exclusion criteria: (1) Patients who have received treatment with non-steroidal anti-inflammatory drugs, cartilage protectants, or adrenal corticosteroids within the past 3 days. (2) Patients with acute or chronic infections, a history of gout, malignancy, and knee joint surgery. This study was approved by the Ethics Committee of West China Hospital, Sichuan University, and all participants signed informed consent forms.

### Baseline data and serum samples

Clinical data, including gender, age, BMI, smoking status, alcohol consumption, K-L classification, diabetes, hypertension, and WOMAC scores, were collected for both groups of subjects. On the day following enrolment, all participants provided a 5 mL venous blood sample on an empty stomach. Blood samples were centrifuged at 3000 r/min for 10 min and stored at -80 °C for subsequent experimental use.

### Cell culture and stimulation

Human normal chondrocyte C28/I2 cell line (YaJi Biological, Shanghai, China) was cultivated in a constant temperature incubator at 37℃, which contained 95% air and 5% carbon dioxide. The cell culture medium consists of RPMI-1640 supplemented with 10% FBS and 1% penicillin/streptomycin. In order to identify the optimal conditions for inducing IL-1β-mediated degeneration of C28/I2 cells, this study first conducted preliminary experiments using concentration and time gradients, ultimately determining that C28/I2 cells were stimulated with 10 ng/mL of IL-1β (MCE, Shanghai, China) for 24 h.

### Cell transfection

MiR-4281 inhibitors/mimics, small interfering RNA (siRNA) targeting BCL3 (si-BCL3), and negative controls (inhibitors-NC, mimics-NC and si-NC) were bought from ThermoFisher (Waltham, USA). Lipofectamine 3000™ (ThermoFisher, USA) was used to transfect plasmids into C28/I2 cells.

### RT-qPCR

Total RNA was extracted using the Trizol method. The extracted total RNA was reverse transcribed using a RT Master Mix for qPCR II (MCE, Shanghai, China). The reverse transcription system was configured as follows: 5 µL of total RNA, 10 µL of 2×miRNA RT Reaction Buffer, 2 µL of miRNA RT Enzyme Mix, and 3 µL of RNase-free ddH_2_O. Follow the instructions in the RT-PCR kit manual to perform the following steps. Prepare the reaction mixture as follows: 2× miRcutting Plus miRNA PreMix 10µL, Forward Primer 0.5µL, Reverse Primer 0.4 µL, 2 µL of first-strand miRNA cDNA, 1.6 µL of 50× ROX Reference Dye, and 5.5 µL of ddH₂O. GAPDH serves as the reference gene for SOX9, COL2A1, MMP3, MMP13 and BCL3, while U6 acts as the reference gene for miR-4281. The relative expression levels of mRNA were calculated using the 2^−ΔΔCt^ method. All primer sequences are shown in Supplementary Table 1.

### Western blot

Western blot analysis was performed to assess the protein expression levels of SOX9, COL2A1, MMP3 and MMP13. C28/I2 cells from each group were harvested and lysed to extract total protein, followed by SDS-PAGE electrophoresis, membrane transfer and blocking. The membranes were then incubated with primary antibodies against SOX9, COL2A1, MMP3 and MMP13, and subsequently with HRP-conjugated secondary antibodies. Protein bands were detected using ECL chemiluminescence reagents, with GAPDH as the internal control. Band intensities were quantified using ImageJ software.

### Cell apoptosis assay

Cells were washed with ddH₂O_2_ at a 1:3 ratio, then resuspended in binding buffer after digestion.

The cells were incubated at room temperature in the dark with 5 µL Annexin V-FITC and 10 µL PI (Merck KGaA, Darmstadt, Germany) for 15 min. After adding 400 µL of PBS, the sample was detected using a flow cytometer (ThermoFisher, USA). Finally, the FlowJo software was used for data analysis to calculate the apoptosis rate of the cells.

### Screening of BCL3

Using miRDB (https://mirdb.org/), the target genes of miR-4281 were obtained with a cutoff threshold of Target Score ≥ 60. The target genes of miR-4281 were obtained from the TargetScan (https://www.targetscan.org/vert_80/) database using the average value of the Total context + + score as the cutoff threshold. In the GeneCards (https://www.genecards.org/) database, relevant disease targets were obtained by using “osteoarthritis” as the search term and setting the cutoff threshold at a Relevance score greater than 10 times the average score. Finally, a Venn diagram was drawn using the Venny online data platform.

### Dual-luciferase assay

Wild-type (BCL3-WT) and mutant (BCL3-MUT) gene vectors of BCL3 were prepared using the pmirGLO (Zhifang, Shanghai, China) plasmid vector. Then, they were co-transfected with miR-4281 inhibitor/mimics into the cells using Lipofectamine 3000™ (ThermoFisher, USA). After 48 h of cultivation, the relative luciferase activity was detected.

### Statistical analysis

Data statistical analysis was conducted using SPSS 26.0 software, with results expressed as mean ± SD. The graphs were created using GraphPad Prism 9 software. For comparison between the two groups, the t-test was used; for comparisons among multiple groups, one-way analysis of variance was employed, and further pairwise comparisons were conducted using Tukey’s post-hoc test. *n* = 3 independent biological experiments, with 3 technical replicates per experiment. *p* < 0.05 indicates statistical significance.

## Result

### The upregulation of miR-4281 in the serum of KOA patients and its diagnostic value

**Fig. 1:**
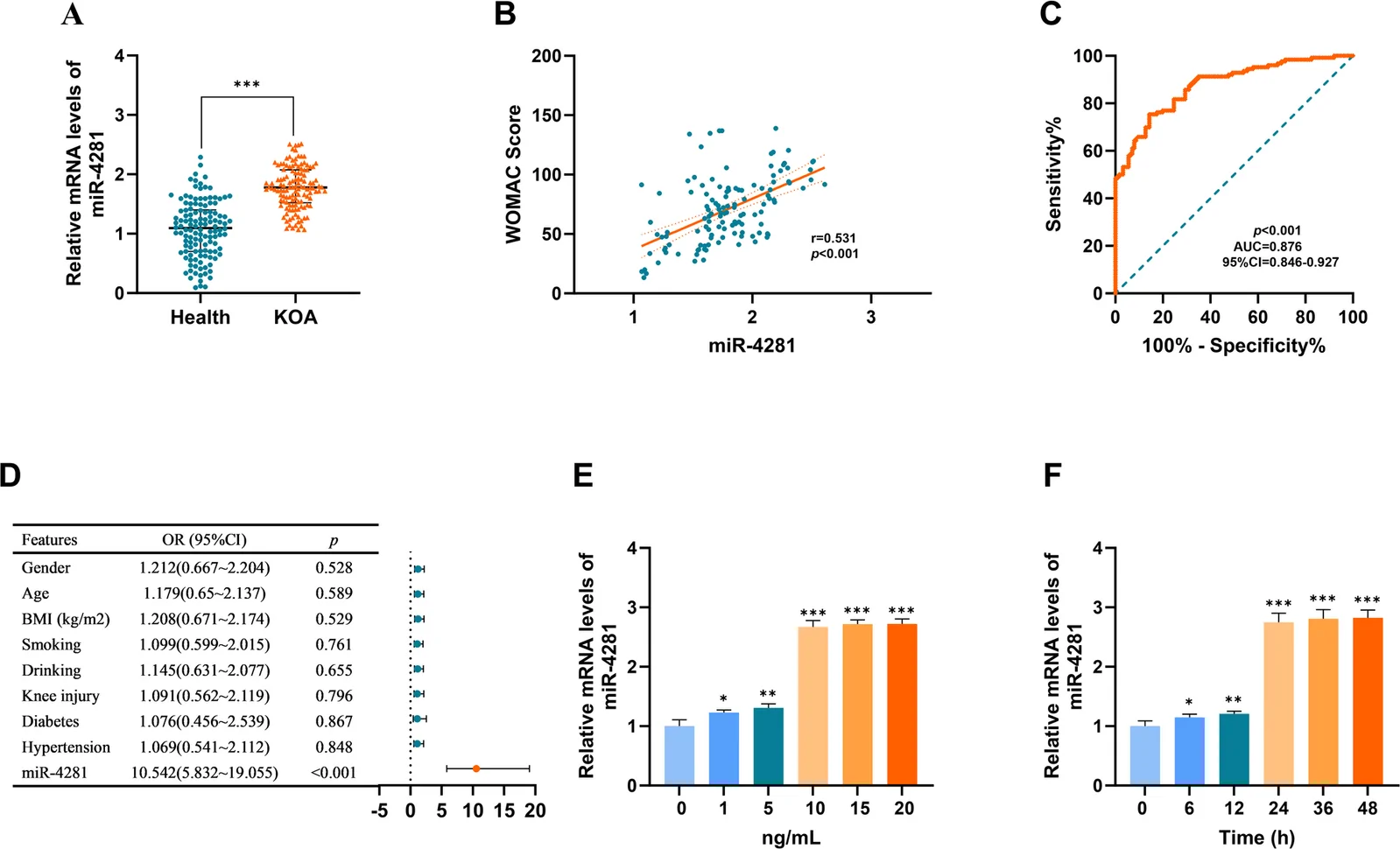
The expression of miR-4281 and its diagnostic value. **A **The miR-4281 expression was upregulated in the serum of KOA patients. **B** miR-4281 was positively correlated with the WOMAC score. **C** The ROC curve of miR-4281 for KOA. **D** Multivariate logistic analysis was used to evaluate the correlation between clinical factors and KOA. **E** The expression of miR-4281 after treatment of C28/I2 cells with different concentrations of IL-1β. **F **The expression of miR-4281 after different durations of treatment with 10 ng/mL IL-1β on C28/I2 cells. Data are presented as mean ± SD. *n* = 3 independent biological experiments, with 3 technical replicates per experiment. Statistical analysis was performed using one-way ANOVA followed by Tukey’s post-hoc test. *p < 0.05, **p < 0.01, ***p < 0.001

By comparing the collected clinical data, it was found that there were no significant differences in gender, age, BMI, smoking, drinking, history of knee trauma, hypertension and diabetes between the two groups of patients (Table 1). RT-qPCR results demonstrated that miR-4281 expression levels in serum samples from KOA patients were significantly elevated compared to the healthy control group (Fig. 1A). Furthermore, the correlation analysis indicated that the expression level of miR-4281 was positively correlated with the WOMAC Score (*r* = 0.531, *p* < 0.001, Fig. 1B). The above results indicate that miR-4281 is crucial for the progression of KOA and may serve as a biomarker for diagnosing KOA. ROC curve analysis indicated an area under the curve (AUC) of 0.905, with a 95% CI ranging from 0.846 to 0.927 (Fig. 1C). Furthermore, at a diagnostic threshold of 1.397, the ROC curve demonstrated a sensitivity of 85.7% and a specificity of 75.4% (Fig. 1C). This ROC curve, along with the corresponding AUC, sensitivity, and specificity results, collectively indicate that this diagnostic model has excellent diagnostic efficacy and performs well in identifying positive cases and excluding negative cases. A binary logistic analysis was conducted to examine the association strength between miR-4281 and other clinical variables and the risk of the disease. The results showed that the OR value of miR-4281 was 10.542 (95% CI: 5.832–19.055), and *p* < 0.001, indicating that it may play an important role in the occurrence of the disease and can be used as a potential biomarker for risk stratification or early screening (Fig. 1D). Following treatment of C28/I2 cells with varying concentrations of IL-1β, the expression of miR-4281 exhibited a concentration-dependent increase. At a concentration of 10 ng/mL, its expression level reached a maximum and stabilised (Fig. 1E). Next, after treating C28/I2 cells with 10 ng/mL IL-1β for different periods of time, it was found that the expression of miR-4281 increased in a time-dependent manner. When the treatment duration reached 24 h, its expression level reached the highest and tended to stabilise (Fig. 1F). Therefore, we chose to use 10 ng/mL of IL-1β to treat the C28/I2 cells for 24 h to establish the KOA cell model.

**Table 1:** The clinical data statistics of the researchers

features	Health (*n* = 126)	KOA (*n* = 126)	*p*
Gender			0.701
Male	53	50	
Female	73	76	
Age			0.611
≤ 60	56	52	
>60	70	74	
BMI (kg /m^2^)			0.705
≤ 23.9	67	64	
>23.9	59	62	
Smoking			0.798
No	73	75	
Yes	53	51	
Drinking			0.450
No	66	60	
Yes	60	66	
History of knee injury			0.570
No	94	90	
Yes	32	36	
Kellgren-Lawrence			-
Ⅱ	-	54	
Ⅲ-Ⅳ	-	72	
Diabetes			0.461
No	111	107	
Yes	15	19	
Hypertension			0.770
No	96	94	
Yes	30	32	
WOMAC Score	-	68.45 ± 26.33	-

### miR-4281 affected the degradation of ECM and apoptosis in C28/I2 cells induced by IL-1β

**Fig. 2:**
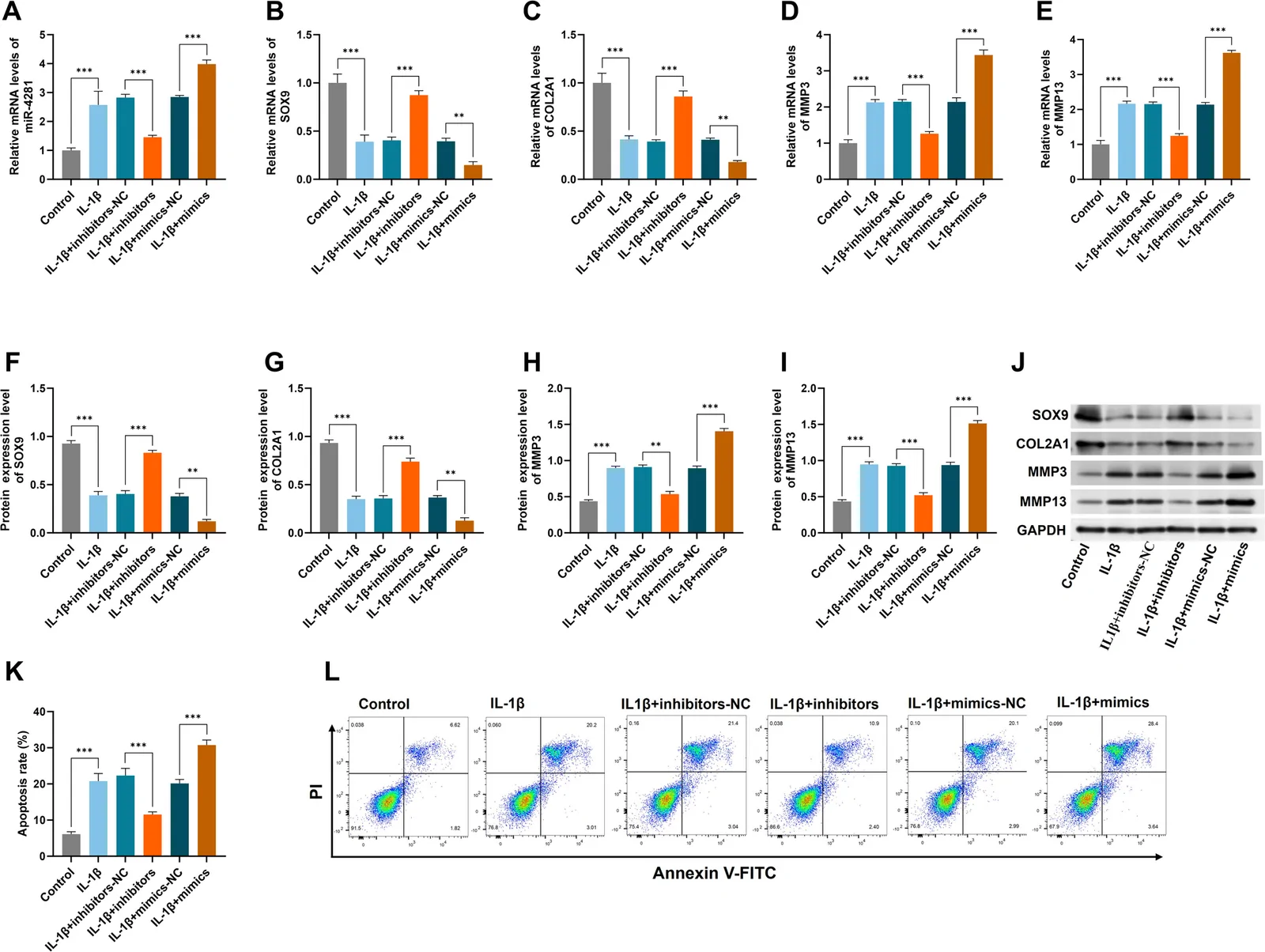
miR-4281 affected the degradation of ECM and apoptosis in C28/I2 cells induced by IL-1β. **A** RT-qPCR was employed to evaluate the transfection efficiency of miR-4281 inhibitors or miR-4281 mimics. **B** and **C** The effects of miR-4281 inhibition or overexpression on IL-1β-induced SOX9 and COL2A1 expression. **D** and **E** The effects of miR-4281 inhibition or overexpression on IL-1β-induced MMP3 and MMP13 expression. **F**-**J** The effects of miR-4281 inhibition or overexpression on IL-1β-induced SOX9, COL2A1, MMP3 and MMP13 protein expression levels. **K** and **L** The effects of miR-4281 inhibition or overexpression on IL-1β-induced apoptosis. Data are presented as mean±SD. *n* = 3 independent biological experiments, with 3 technical replicates per experiment. Statistical analysis was performed using one-way ANOVA followed by Tukey’s post-hoc test. ***p < 0.001

To elucidate the potential mechanism by which miR-4281 influences KOA, miR-4281 inhibitors or miR-4281 mimics were successfully transfected into IL-1β-induced C28/I2 cells. The RT-qPCR results showed that the exposure to IL-1β significantly increased the level of miR-4281, and the miR-4281 inhibitors partially reversed this increase, while the miR-4281 mimics further increased the level of miR-4281. (Fig. 2A). Analysis of SOX9 and COL2A1 revealed that exposure to IL-1β significantly reduced the expression of both genes, whereas miR-4281 inhibitors largely restored their expression levels; conversely, miR-4281 mimics further reduced their levels (Fig. 2B and C). More importantly, analysis of MMP3 and MMP13 revealed that exposure to IL-1β significantly increased their expression levels, whereas miR-4281 inhibitors largely restored the expression of MMP3 and MMP13; conversely, miR-4281 mimics further increased their expression levels (Fig. 2D and E). It is worth noting that the western blot results shown in Fig. 2F and J have provided a strong validation of the aforementioned findings at the protein level. Finally, the effect on apoptosis of C28/I2 cells was evaluated. The results showed that exposure to IL-1β significantly increased the apoptosis level of C28/I2 cells, while miR-4281 inhibitors partially reversed this increase; conversely, miR-4281 mimics further promoted apoptosis (Fig. 2K and L).

### The proof of the relationship between miR-4281 and BCL3

**Fig. 3:**
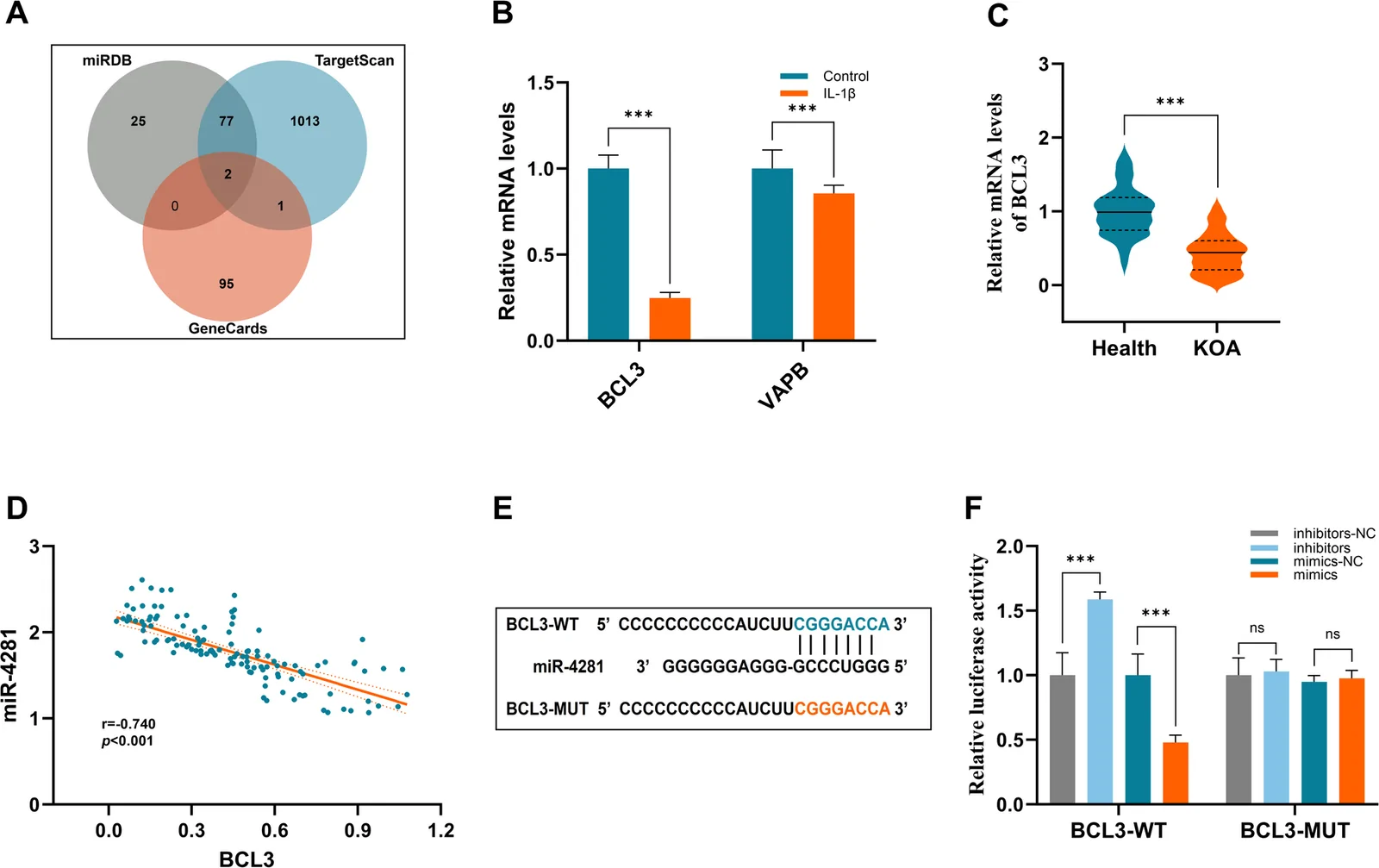
miR-4281 targeted BCL3. **A** Venn diagram for screening miR-4281 downstream targets. **B** The expression levels of BCL3 and VAPB after IL-1β stimulation of C28/I2 cells. **C** The BCL3 expression was downregulated in the serum of KOA patients. **D** The expression levels of miR-4281 and BCL3 are negatively correlated. **E** The binding site diagram of miR-4281 with the 3’UTR of BCL3. **F** The dual-luciferase assay demonstrated that miR-4281 targets BCL3. “ns” indicates no statistically significant difference. Data are presented as mean ± SD. *n* = 3 independent biological experiments, with 3 technical replicates per experiment. Statistical analysis was performed using one-way ANOVA followed by Tukey’s post-hoc test. ***p < 0.001

The miRDB and TargetScan databases independently predicted 104 and 1093 downstream targets of miR-4281, respectively, while the GeneCards database retrieved 98 disease targets related to osteoarthritis (Fig. 3A). The Venn diagram indicates that BCL3 and VAPB are common genes among the three. They are most likely downstream targets regulated by miR-4281. The RT-qPCR results indicated that IL-1β stimulation led to a significant downregulation of BCL3, while VAPB showed a slight decrease (Fig. 3B). Therefore, BCL3 was selected as a candidate target for investigation among the downstream genes of miR-4281. First, the expression level of BCL3 in the serum was analysed. The results showed that, compared with healthy individuals, the expression level of BCL3 in the KOA was abnormally decreased (Fig. 3C). It is noteworthy that correlation analysis indicates a negative regulatory relationship between miR-4281 and BCL3 (*r*=-0.740, *p* < 0.001, Fig. 3D). Figure 3E presents the binding sites and mutation sites of miR-4281 and BCL3. More importantly, the results of the dual luciferase assay indicated that in C28/I2 cells containing the wild-type BCL3 plasmids, miR-4281 inhibitors significantly enhanced the fluorescence activity, while miR-4281 mimics significantly weakened this activity (Fig. 3F). In cells containing mutant-type BCL3 plasmids, miR-4281 inhibitors/mimics did not affect the fluorescence activity (Fig. 3F). The above results collectively demonstrate the negative regulatory relationship between miR-4281 and BCL3.

### miR-4281 influenced ECM and apoptosis in IL-1β-induced C28/I2 cells via BCL3

**Fig. 4:**
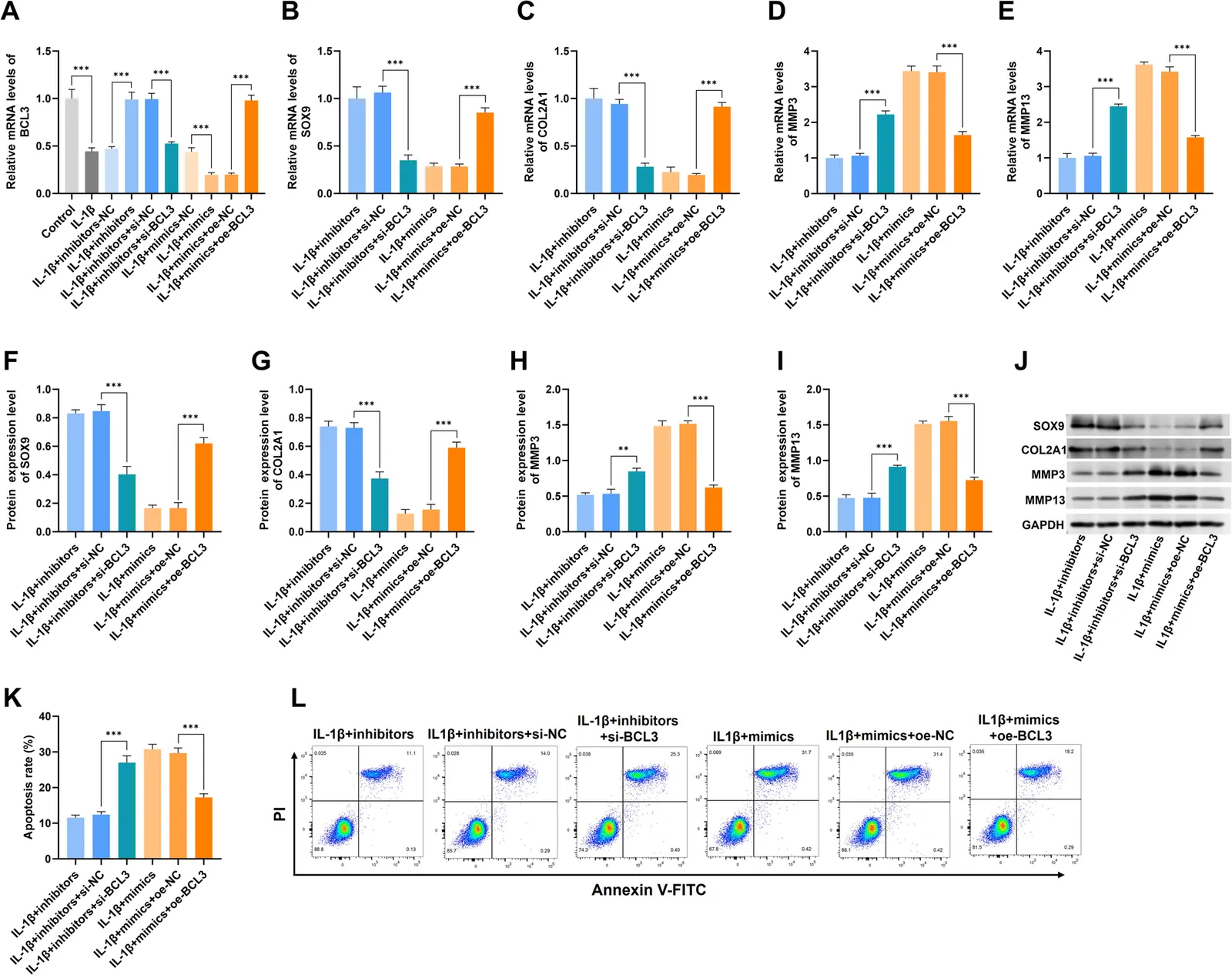
miR-4281 influenced ECM and apoptosis in IL-1β-induced C28/I2 cells via BCL3. **A** RT-qPCR was employed to evaluate the co-transfection efficiency of miR-4281 inhibitors and si-BCL3 or miR-4281 mimics and oe-BCL3. **B** and **C **BCL3 reversed the effect of miR-4281 on SOX9 and COL2A1. **D** and **E** BCL3 reversed the effect of miR-4281 on MMP3 and MMP13. **F** - **J** BCL3 reversed the effect of miR-4281 on the protein expression levels of SOX9, COL2A1, MMP3 and MMP13. **K** and **L** BCL3 reversed the apoptosis induced by miR-4281. Data are presented as mean ± SD. *n* = 3 independent biological experiments, with 3 technical replicates per experiment. Statistical analysis was performed using one-way ANOVA followed by Tukey’s post-hoc test. ***p < 0.001

To further investigate the specific mechanism by which miR-4281 influences KOA progression through regulating chondrocyte ECM and apoptosis, miR-4281 inhibitors and si-BCL3 or miR-4281 mimics and oe- BCL3 were successfully co-transfected into IL-1β-induced C28/I2 cells. RT-qPCR revealed that IL-1β stimulation markedly suppressed BCL3 expression, while miR-4281 inhibitors significantly upregulated its expression. Co-inhibition of miR-4281 and BCL3 reversed the miR-4281 inhibitor-induced increase in BCL3 levels; conversely, miR-4281 mimics significantly downregulated BCL3 expression, and co-overexpression of miR-4281 and BCL3 reversed the suppression of BCL3 levels by miR-4281 mimics (Fig. 4A). The subsequent results of the cell function experiments revealed the effects of BCL3 on the ECM and apoptosis of chondrocytes. Analysis of SOX9 and COL2A1 revealed that silencing BCL3 reversed the increase in SOX9 and COL2A1 expression induced by miR-4281 inhibitors, while overexpression of BCL3 reversed the suppression of SOX9 and COL2A1 expression caused by miR-4281 mimics (Fig. 4B and C). Analysis of MMP3 and MMP13 revealed that silencing BCL3 reversed the reduction in their expression caused by miR-4281 inhibitors, while overexpression of BCL3 reversed the increase in their expression caused by miR-4281 mimics (Fig. 4D and E). It is worth noting that the western blot results shown in Fig. 2F and J have provided a strong validation of the aforementioned findings at the protein level.

More importantly, the expression levels of these four molecules at the protein level were consistent as determined by RT-qPCR (Fig. 4F and J). Finally, the assessment of cell apoptosis revealed that silencing BCL3 reversed the inhibitory effect of miR-4281 on the apoptosis of C28/I2 cells induced by IL-1β, whereas overexpression of BCL3 produced the opposite effect (Fig. 4K and L).

## Discussion

The core pathological process of KOA is the degeneration of articular cartilage. A vicious cycle of net loss of ECM, triggered by metabolic imbalance in chondrocytes, serves as the key driver propelling disease progression [20]. The post-transcriptional regulation mediated by miRNAs plays a crucial role in this process, forming a vicious pathological cycle of “metabolism - epigenetics - ECM degradation”. This study conducted clinical and cellular experiments focusing on miR-4281, further verifying the core role of this circulating molecule, clarifying the specific mechanism by which miR-4281 targets BCL3 and participates in the pathological process of KOA, and confirming its potential diagnostic value.

Clinical data from this study indicate that serum miR-4281 expression levels were significantly elevated in KOA patients, exhibiting a positive correlation with WOMAC scores. ROC curve analysis confirms its high diagnostic efficacy for KOA. This is consistent with the existing reports that miR-146b, miR-27b and miR-338-3p are biomarkers for the early diagnosis of osteoarthritis [21, 22]. Together, they provide strong data support for miRNAs to serve as non-invasive diagnostic markers for KOA.

From the perspective of the pathological pathway, miR-4281, as an epigenetic regulatory molecule, its abnormally high expression directly disrupts the homeostasis of the synthesis and degradation metabolic processes of chondrocytes, triggering and exacerbating the vicious cycle of “metabolism - epigenetics - ECM degradation”. Specifically, as a key transcription factor governing chondrocyte differentiation and ECM synthesis, the reduced expression of SOX9 constitutes the initiating step in the suppression of ECM anabolism within KOA [23]. In this study, in the KOA cell model induced by IL-1β, the high expression of miR-4281 was accompanied by a significant downregulation of SOX9 expression. After transfection with the miR-4281 inhibitor, the expression of SOX9 significantly recovered, suggesting that miR-4281 may inhibit the synthesis and metabolism of ECM by suppressing the expression of SOX9. Within the ECM anabolic suppression pathway, reduced levels of SOX9 directly result in downregulation of its downstream target gene COL2A1. This diminished expression inevitably leads to insufficient synthesis of type II collagen [24]. Type II collagen, as the core structural component of the ECM, is crucial for maintaining the tensile strength and structural integrity of cartilage tissue [25]. Its reduced synthesis compromises the structural integrity of cartilage tissue, laying the groundwork for net loss of the extracellular matrix. Meanwhile, the abnormally high expression of miR-4281 also significantly activated the catabolic pathways of chondrocytes. In IL-1β-induced C28/I2 cells, high expression of miR-4281 was accompanied by a significant upregulation of MMP3 and MMP13 expression, whereas the miR-4281 inhibitor effectively suppressed the expression of both. This result is consistent with the conclusion from previous studies that “the MMP family (especially MMP3 and MMP13) is the core proteases for ECM degradation” [26]. MMP3 primarily degrades proteoglycans within the cartilage matrix, whilst MMP13 specifically degrades type II collagen [27]. The synergistic overexpression of both enzymes accelerates the degradation rate of type II collagen and proteoglycans, further exacerbating the loss of ECM components. As mentioned above, the inhibition of anabolic metabolism and the activation of catabolic metabolism form a synergistic effect, ultimately leading to the net loss of ECM. This phenomenon is consistent with the effect of miR-4281 on apoptosis of C28/I2 cells as observed in this study.

In terms of mechanism, this study has confirmed that miR-4281 can directly target and regulate the expression of BCL3. This finding has clarified the specific molecular target of miR-4281 in the pathological process of KOA. Furthermore, this study demonstrated through cell functional experiments that miR-4281 regulates the pathological process of KOA, mainly by targeting and inhibiting BCL3. When miR-4281 is highly expressed, BCL3 expression is suppressed, thereby inhibiting the synthesis of SOX9 and COL2A1 while promoting the expression of degradative enzymes such as MMP3 and MMP13. Concurrently, it induces chondrocyte apoptosis, exacerbating the net loss of the ECM and accelerating cartilage degeneration. Following suppression of miR-4281 expression, BCL3 expression recovers, promoting anabolic processes while inhibiting catabolic activity. Concurrently, it suppresses chondrocyte apoptosis, alleviates ECM degradation and cartilage damage, thereby disrupting the vicious cycle of “metabolism-epigenetics-ECM degradation”. However, overexpression of miR-4281 has the opposite effect. The findings of this study are also in line with those of previous research. For instance, a study has shown that miR-146b participates in the pathological process of KOA by regulating the enzymes related to ECM degradation [28]. Another study also demonstrated that miR-33b-3p targets DNMT3A and promotes the proliferation of chondrocytes, inhibits the apoptosis of chondrocytes and the degradation of cartilage ECM [29]. This study has for the first time, identified miR-4281 targeting BCL3 as a participant in the vicious cycle of “metabolism-epigenetics-ECM degradation” in KOA, thereby filling a research gap concerning miR-4281 in KOA. Furthermore, as a member of the nuclear IκB family, BCL3 has been shown to be involved in regulating the NF-κB pathway, which is associated with inflammation and metabolism, and plays a key role in maintaining chondrocyte homeostasis [30]. During the pathogenesis of osteoarthritis, BCL3 expression is significantly downregulated, weakening its inhibitory effect on the NF-κB pathway; this, in turn, promotes the expression of inflammatory factors and matrix-degrading enzymes, thereby exacerbating cartilage degeneration [31]. The results of this study indicate that BCL3, as a direct downstream target of miR-4281, is negatively regulated by miR-4281; bidirectional rescue experiments further confirm that BCL3 acts as a key mediator in the regulation of SOX9, COL2A1 and MMP3/13 expression, as well as apoptosis, by miR-4281. Based on the existing literature, we hypothesise that BCL3 may maintain the metabolic balance of cartilage by inhibiting the NF-κB pathway, thereby upregulating SOX9-mediated anabolic processes in cartilage whilst simultaneously suppressing MMP3/13-mediated catabolic processes. Although this study did not directly assess the activation status of the relevant signalling pathways, this regulatory model is indirectly supported by all the phenotypic data obtained in this study. Future research will focus more closely on the specific signalling pathways regulated by BCL3 to elucidate its mechanism of action in OA in greater detail. In conclusion, the clarification of this mechanism not only improves the molecular network by which miRNAs regulate the pathological process of KOA, but also provides a new idea for the targeted treatment of KOA - by inhibiting the expression of miR-4281 or enhancing the expression of BCL3, it may become an effective strategy for intervening in the cartilage degeneration of KOA.

The clinical samples in this study were all sourced from a single centre, which introduces a degree of selection bias. Furthermore, the relevant functional and mechanistic experiments were conducted solely using in vitro chondrocyte cell lines and have not yet been validated in animal models of osteoarthritis. Additionally, this study did not examine BCL3-downstream signalling pathways at the protein level, and the detailed transcriptional regulatory network remains to be explored in future research.

## Conclusion

In summary, this study confirms that miR-4281 possesses good diagnostic value for KOA. miR-4281 inhibitors upregulated the expression of BCL3, thereby promoting the expression of SOX9 and COL2A1, while inhibiting the expression of MMP3 and MMP13 as well as cell apoptosis. Eventually, it inhibits cartilage degeneration and the progression of KOA.

## Supplementary Information

Supplementary Material 1.

## Data Availability

The datasets used and/or analysed during the current study are available from the corresponding author on reasonable request.
