# Supplementary material for: The diagnostic value of miR-4281 for knee osteoarthritis and its mechanism of action on the extracellular matrix of chondrocytes and apoptosis
Source: Hereditas. 2026 Jul 25;163:118. doi: 10.1186/s41065-026-00711-9 (PMC13688781; doi:10.1186/s41065-026-00711-9)
Supplement: Supplementary file 1 [file 41065_2026_711_MOESM1_ESM.docx]

Supplementary Table 1: The primer sequences for RT-qPCR.

| Gene | Sequences (5’-3’) |
| --- | --- |
| miR-4281 | F: AAGAATTGGGTCCCGGGGAG |
|  | R: CAGTGCAGGGTCCGAGGT |
| SOX9 | F: GTCGGTGAAGAATGGGCAAG |
|  | R: GACCCTGAGATTGCCCGGA |
| COL2A1 | F: ATGATTCGCCTCGGGGCTC |
|  | R: CACAGACACAGATCCGGCA |
| MMP3 | F: GAGGACACCAGCATGAACCT |
|  | R: CACCTCCAGAGTGTCGGAGT |
| MMP13 | F: AACCGCAGCACTGAGCCTTTTC |
|  | R: ATACGAGCATCCATCCCGAGACC |
| BCL3 | F: CCGGAGGCCCTTTACTACCA |
|  | R: GGAGTAGGGGTGAGTAGGCAG |
| GAPDH | F: GTCAAGGCTGAGAACGGGAA |
|  | R: AAATGAGCCCCAGCCTTCTC |
| U6 | F: CTCGCTTCGGCAGCACATATACT |
|  | R: ACGCTTCACGAATTTGCGTGTC |
